# FeNi decorated nitrogen-doped hollow carbon spheres as ultra-stable bifunctional oxygen electrocatalyst for rechargeable zinc–air battery with 2.7% decay after 300 hours cycling†

**DOI:** 10.1039/d3ra08572d

**Published:** 2024-01-25

**Authors:** Shengjie Lun, HanBin Wang, Yijing Deng, Jinting Cui, Pei Liang, Kaiwen Wang, Lin Lv, Houzhao Wan, Hao Wang

**Affiliations:** a Hubei Yangtze Memory Laboratories Wuhan 430205 China hanbinwang@hubu.edu.cn nanoguy@126.com; b School of Microelectronics, Hubei University Wuhan 430062 China; c College of Optical and Electronic Technology, China Jiliang University Hangzhou 310018 China plianghust@gmail.com

## Abstract

Research on non-noble metal bifunctional electrocatalysts with high efficiency and long-lasting stability is crucial for many energy storage devices such as zinc–air batteries. In this report, nitrogen-doped porous hollow carbon spheres with a size of about 300 nm were fabricated using a modified Stöber method and decorated with an FeNi alloy through a pyrolytic reduction process, resulting in a promising bifunctional electrocatalyst for both the oxygen evolution reaction and oxygen reduction reaction. The as-prepared FeNi@NHCS electrocatalyst exhibits excellent bifunctional activity in KOH electrolyte, attributed to its mesoporous structure, large specific surface area, and the strong coupling between the FeNi nanoalloy and nitrogen-doped carbon carriers. The electrocatalyst demonstrates excellent ORR performance with *E*_1/2_ = 0.828 V and OER activity with *E*_*j*=10 mA_ = 1.51 V. A zinc–air battery using FeNi@NHCS as the air electrode achieves an open-circuit voltage of 1.432 V and a maximum power density of 181.8 mW cm^−2^. After 300 h of galvanostatic charge–discharge cycles, the charge–discharge voltage gap (Δ*U*) of the battery had only decayed by 2.7%, demonstrating superior cycling stability.

## Abstract 2

The Zn–air battery assembled by the bifunctional FeNi@HMCS electrocatalyst displayed a maximum power density of 181.8 mW cm^−2^ and superior stability for over 300 hours.

## Graphical abstract

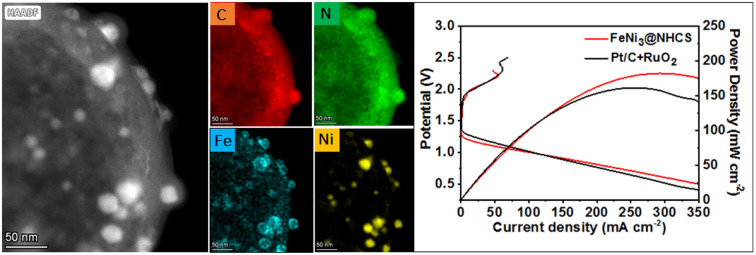

## Introduction

The consumption of fossil fuels and increasing environmental pollution have made it imperative to develop green and renewable energy storage and conversion technologies.^1,2^ Rechargeable zinc–air batteries (ZABs) are promising devices for energy storage and conversion, due to their high energy density (1086 W h g^−1^), low cost, eco-friendliness, and intrinsic safety.^3,4^ However, the electrochemical performance of ZABs is hindered by the slow kinetics of the oxygen reduction reaction (ORR) and oxygen evolution reaction (OER) during discharging/charging processes at cathodes, limiting the overpotential and energy conversion efficiency of ZABs.^5^ There is a pressing need to develop stable and effective bifunctional electrocatalysts for rechargeable ZABs. Conventional noble metal materials, such as Pt and Ir/Ru oxide electrocatalysts, are currently employed as state-of-the-art catalysts for ORR and OER, respectively. However, these materials face several drawbacks, including high cost, scarcity, limit stability, and insufficient bifunctional catalytic activity, which severely restrict the practical application of ZABs.^6,7^ Consequently, it is critically important to rationally design and fabricate noble metal-free bifunctional electrocatalysts that exhibit high efficiency and excellent durability.^8–10^

Over the past decade, many efforts have been devoted to develop non-noble metal bifunctional electrocatalysts, such as oxides,^11–14^ transition metals and their alloys,^15–18^ sulphides,^19^ nitrides,^20,21^ and various carbon materials.^22,23^ Among these, the transition metal–nitrogen/carbon compound often exhibited excellent electrocatalytic activity and long-term stability for OER/ORR. So far, Fe–N_*x*_–C catalysts are considered as efficient ORR catalysts due to their high intrinsic activity, which is achieved by introducing transition metal into the nitrogen-doped carbon matrix, optimizing its electronic structure as well as the adsorption free energy of surface intermediates (*e.g.*, OOH*, O*, OH*).^24,25^ For example, Wu *et al.* discovered Fe–N_*x*_–C nanofiber catalyst with superior ORR performance, showing a positive onset of 0.94 V and a half-wave potentials of 0.82 V, respectively.^26^ Zong *et al.* developed a cost-efficient synthetic approach to obtain Fe/Fe_3_C@Fe–N–C bifunctional electrocatalysts, demonstrating a maximum power density of 147 mW cm^−2^ with a narrow discharge/charge voltage gap of 0.87 V.^27^ However, the generation of semiconductive FeOOH in Fe–N–C materials during OER inhibited their activity and electron transfer. Moreover, the weak binding strength of FeOOH also inactivated the Fe site.^28,29^ A feasible strategy to optimize the OER activity of Fe–N–C materials involves hybridizing them with other transition metals, such as Ni and Co. The lattice-oxygen-mediated mechanism (LOM) introduces the concept of synergistic catalysis by adjacent bimetallic sites and considers metal alloys as OER reaction sites.^30,31^ Inspired by this mechanism, FeNi alloys have garnered significant attention due to their excellent conductivity and flexible electronic structure. Experimentally, Wu *et al.* successfully embedded FeNi alloys in N-doped carbon with custom structures by introducing the MOF precursor and polymer coating/packaging strategy. The solid-state Zn–air battery made by FeNi@carbon nanotubes displayed an open circuit voltage of 1.38 V and a maximum power density of 81 mW cm^−2^.^32^ Zheng *et al.* synthesized FeNi nanoparticles embedded nitrogen-doped carbon nanotubes using cotton pads as the substrate. These nanotubes exhibited a high half-wave potential of 0.85 V and a low potential of 1.59 V at 10 mA cm^−2^.^33^ Liu *et al.* fabricated a series of FeNi@N-graphite core–shell nanostructures that demonstrated their high performance as multifunctional catalysts for OER, ORR, and HER. The assembled Zn–air battery shows an open-circuit potential of 1.482 V, with superior stability after cycling 40 h at 20 mA cm^−2^.^34^

Along with the alloying strategy, coupling with suitable matrix of electrocatalyst has emerged as an essential step toward the enhancement of its activity and stability. In this regard, the heteroatom-doped carbon materials have demonstrated excellent electrocatalytic ORR performance. Heteroatoms doping promotes many processes, such as polarizing and changing the spin density of adjacent carbon atoms, generating new catalytic active sites and promoting chemical adsorption/desorption of intermediates on the electrocatalyst surface, thus enhancing ORR catalytic activity.^35,36^ Therefore, effectively coupling heteroatom-doped carbon matrix with transition metal alloys is a promising strategy for achieving high-performance bifunctional electrocatalysts.^37,38^ On this basis, the morphology and nanopore control of the carbon carriers would provide more structural advantages, which help to improve the reaction kinetics.^39^

Herein, we successfully design and fabricate a novel FeNi decorated porous carbon nanostructure through a modified Stöber method and subsequent pyrolysis reduction strategy. The porous hollow structure with well-crystallized FeNi nanoparticles attached to the carbon surface help to enhance the charge transfer while facilitate the diffusion/penetration process between the reactant-electrode and electrolyte. Though assembling the FeNi@NHCS as the cathode, the Zn–air battery displayed a large specific capacity of 714 mA h g_Zn_^−1^ (at 10 mA cm^−2^), a maximum power density of 181.8 mW cm^−2^ and superior stability for over 300 hours. The study highlights the advantages of bimetallic nanoparticle decorated NHCS nanostructure in oxygen electrocatalysis, and indicates a new strategy for the development of high-performance rechargeable Zn–air battery.

## Experimental

### Sample preparation

#### Synthesis of NHCS

SiO_2_@dopamine (PDA) spheres were synthesized through a modified Stöber method. In details, the mixture containing deionized water (160 mL), anhydrous ethanol (48 mL), and NH_3_·H_2_O (2 mL) was stirred for 30 min. Then, 2 mL of TEOS was slowly drop into the solution and stirring continued for 15 min at room temperature. Next, deionized water (16 mL) dissolved with 0.8 g dopamine hydrochloride was poured into the mixture and magnetically stirred for 20 h. The product was then centrifuged and washed by three times with 50 mL of deionized water and 50 mL of ethanol alternately. After dried in a 60 °C oven for 12 h, the SiO_2_@PDA was obtained. To carbonize the PDA to nitrogen-doped carbon, the SiO_2_@PDA was annealed at 800 °C for 3 hours under N_2_ (99.99%). The heating rate of the tube furnace was 5 °C per minute before reach to 800 °C. Finally, the SiO_2_@nitrogen doped carbon spheres were prepared. To obtain nitrogen-doped porous hollow carbon spheres (NHCS), the SiO_2_ template was removed by etching the products in 80 mL of NaOH solution (2 M) for 12 h.

#### Synthesis of FeNi@NHCS

The deposition of FeNi alloy on NHCS was conducted through pyrolysis reduction of metal chlorides. In details, 0.027 g of FeCl_3_·6H_2_O and 0.0237 g of NiCl_2_·6H_2_O were dissolved in 20 mL of deionized water and then 30 mg of NHCS was added into the solution. The mixture was stirred for 6 h and then dried at 60 °C in an oven for 14 h. To prepare FeNi@NHCS samples under different annealing temperature (T0–T4), the dried product was heated at 400 °C, 500 °C, 600 °C, 700 °C and 800 °C, respectively, in H_2_/Ar_2_ (5%/95%) atmosphere. In order to regulate the composition of FeNi alloy, samples R1, R2, R3, and R4 were prepared and synthesized using the same method at 400 °C, with the only difference being the molar ratio of the initial Fe and Ni sources.

### Preparation of electrode liquid for test

For the preparation working electrode, 3 mg of FeNi@NHCS powder was dispersed in a mixture of 465 μL DMF and 35 μL Nafion (5 wt%). After 2 h of ultrasound, the uniformly dispersed electrocatalyst ink was obtained. Then, 4 μL electrocatalyst ink was evenly deposited onto a clean glassy carbon electrode and dried in the air. This process was repeated five times. For comparison, the Pt/C and RuO_2_ catalyst electrodes were prepared using the same method.

### Structure and electrochemical characterization

The structure of the NHCS and FeNi@NHCS samples were systematically investigated by widely used structural analysis instruments and methods. Please refer to the ESI† for specific analysis and testing details. The electrochemical properties of FeNi@NHCS samples were measured by electrochemical workstation (CHI 760e) equipped with a rotating disk electrode (RDE) system. The standard three-electrode system was adopted, which consists of a working electrode, a reference electrode (Ag/AgCl electrode), and a counter electrode (platinum wire). All the potentials in the electrochemical test are standardized to the reversible standard hydrogen electrode (RHE).

The cyclic voltammetry (CV) curves were obtained at a scanning rate of 50 mV s^−1^ in nitrogen/oxygen-saturated KOH solution (0.1 M) for ORR/OER measurements. In ORR test, the linear sweep voltammetry (LSV) was performed in oxygen saturated solution from 0.2 V to 1.2 V (*vs.* RHE). The scan rate is 5 mV s^−1^ and the rotating speed is 1600 rpm. In OER test, the LSV was carried out in the nitrogen saturated KOH solution (1 M) from 1.0 V to 2.0 V (*vs.* RHE) with the same scan rate and rotating speed. The Tafel slopes in ORR were obtained according to the relationship between the overpotential and log(*j*_k_). The ORR/OER durability of the electrocatalysts was evaluated by performing a potential cycling of 10 000/3000 cycles between 0.2 V to 1.2 V/1.0 V to 2.0 V (*vs.* RHE) at a scan rate of 50 mV s^−1^, respectively. This accelerated durability test provides insight into the long-term stability and performance of the electrocatalysts under realistic operating conditions. The electron transfer number (*n*) per oxygen molecule during ORR was calculated according to the Koutecky–Levich equation. Details of the calculation were provided in the support information of this work.

### Fabrication and testing of rechargeable Zn–air battery

A polished zinc sheet (3 cm × 1.8 cm) was used as the anode and the electrolyte was made of 6 M KOH and 0.02 M Zn(CH_3_COO)_2_ aqueous solutions. For assembling a liquid zinc–air battery. The air electrode was fabricated using the FeNi@NHCS electrocatalysts, which were loaded onto a composite matrix (2.5 cm × 2 cm) comprising carbon paper, foam nickel, and a waterproof film. The exposed area to the electrolyte was approximately 1 cm^2^, and the loading density of the catalyst was about 2.0 mg cm^−2^. Battery performance measurements were conducted using a BTS 7.6.X battery testing system. The recurrent galvanostatic pulse method was employed to obtain the Galvanostatic charge–discharge curves of the battery, with a current density of 10 mA cm^−2^ and a 20 minute cycle duration (10 minutes for discharge and 10 minutes for charge).

## Results and discussion

**Fig. 1:**
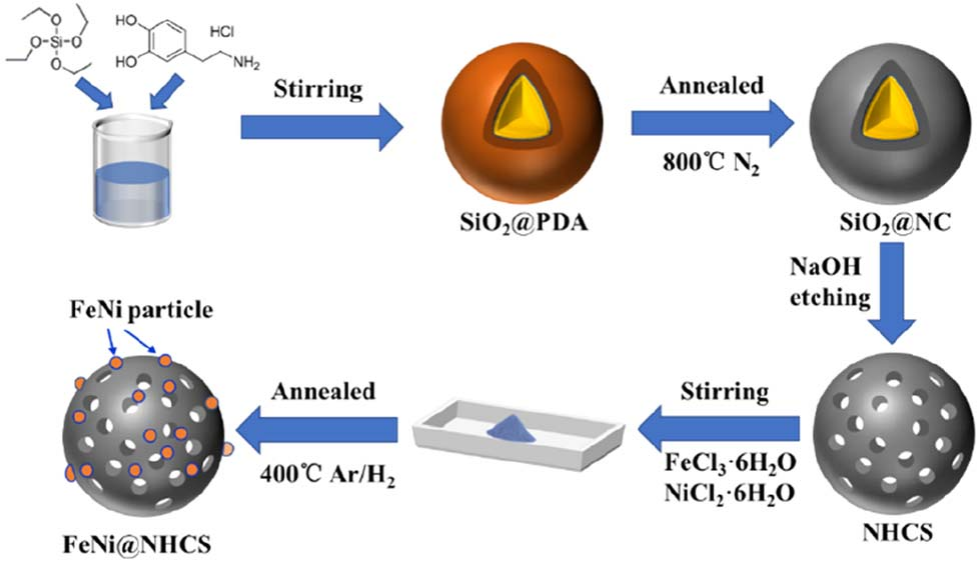
Schematic of the fabrication of FeNi@NHCS.

**Fig. 2:**
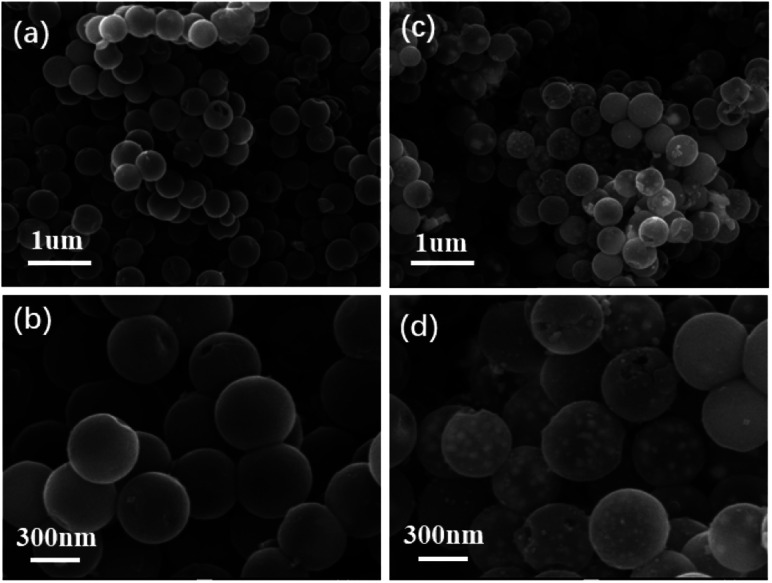
SEM images of (a and b) NHCS sample; (c and d) FeNi@NHCS (sample R2).

The synthesis strategy for FeNi@NHCS is explained in Fig. 1. SiO_2_ microspheres coated with polydopamine were prepared *via* hydrolysis of ethyl orthosilicate (TEOS) and self-polymerization of dopamine (PDA) under alkaline conditions. Subsequently, PDA was carbonized into a carbon shell at high temperature in a N_2_ atmosphere, and the silica template was etched by NaOH solution to obtain nitrogen-doped hollow carbon spheres (NHCS). Finally, metal salt precursors (NiCl_2_·6H_2_O and FeCl_3_·6H_2_O) and NHCS were mixed in a solution and dried to ensure sufficient contact. Pyrolysis reduction was conducted an H_2_/Ar_2_ atmosphere to reduce the metal salts to alloys. For comparison, nitrogen-doped hollow carbons decorated with different Fe/Ni proportions (Fe : Ni = 1 : 2, 1 : 3, 1 : 4, and 1 : 5, respectively) and at various annealing temperatures (400–800 °C) were synthesized and investigated. The morphologies of the as-synthesized NHCS and FeNi_3_@NHCS were characterized by scanning electron microscopy (SEM). As displayed in Fig. 2(a) and (b), the NHCS exhibited hollow spherical morphology with a diameter of approximately 300 nm. Additionally, the cracks and pores exposed on the surface indicate a hollow structure. In Fig. 2(c) and (d), sample R2 displayed a similar morphological structure, with the surface modified by FeNi alloy nanoparticles with 30 nm in size. Notably, the spherical morphology of NHCS remained essentially unchanged after the introduction of FeNi alloy nanoparticles through annealing. This indicates that the prepared NHCS substrate effectively disperse the FeNi alloy nanoparticles, preventing agglomeration. However, as the temperature increased from 400 °C to 800 °C, the FeNi alloy nanoparticles exhibited slight agglomeration, and the cracks and damage on the carbon sphere surface became more pronounced (Fig. S1†).

**Fig. 3:**
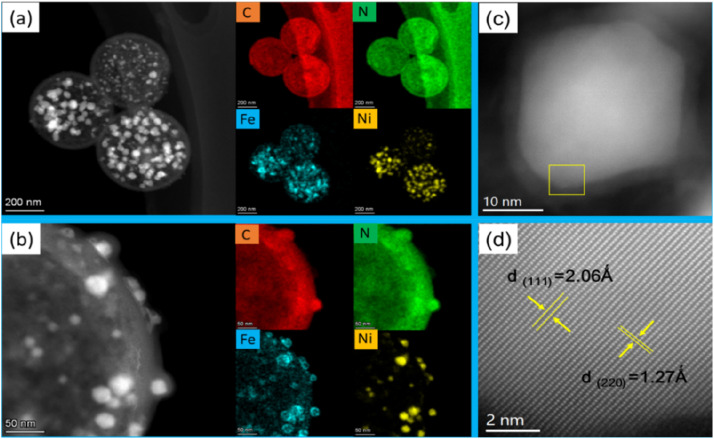
(a) STEM image of the several FeNi@HCMSs with their EELS elemental mapping images of C, N, Fe and Ni; (b) a magnified STEM image of one typical FeNi@HCMS with its elemental EELS mapping images of C, N, Fe and Ni. (c) TEM image of one FeNi nanoparticle (d) high-resolution TEM image of the FeNi nanoparticle marked in (c).

**Fig. 4:**
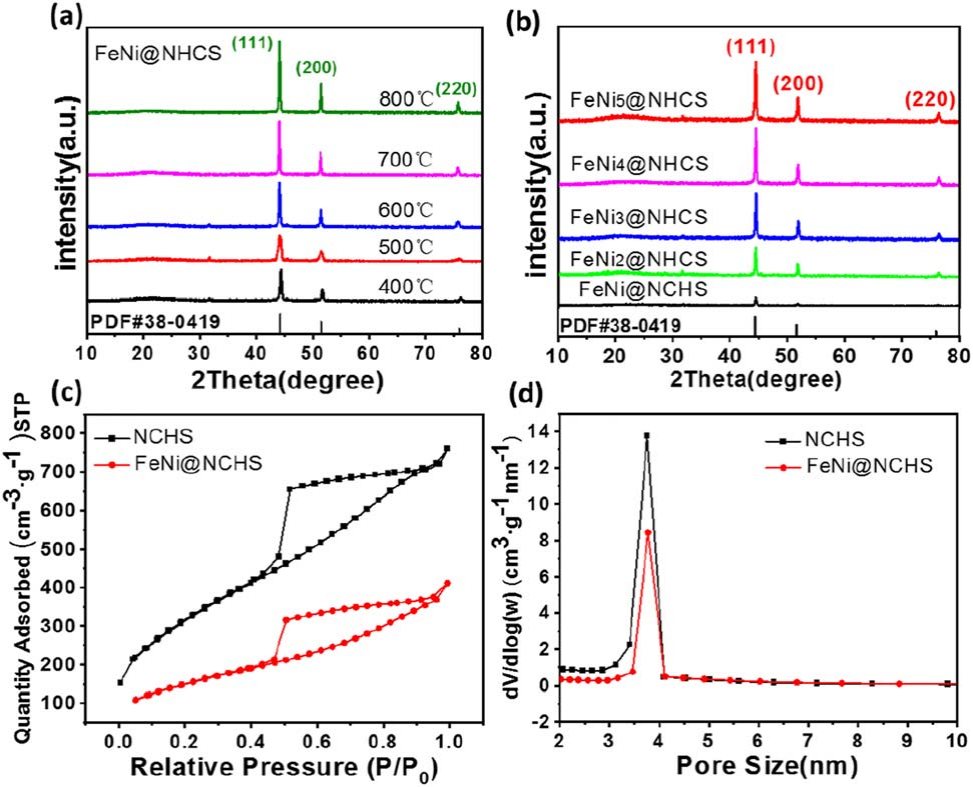
(a and b) XRD patterns of NHCS and sample (R0–R4); (c and d) N_2_ adsorption–desorption isotherms and BJH pore-size distribution curves of NHCS and sample R2.

The microstructure of the FeNi@NHCS sample were further analysed by scanning transmission electron microscopy (STEM). As shown in Fig. 3(a), FeNi nanoparticles with sizes between 5 nm and 30 nm are homogenously deposited on the surface of the NHCS skeleton. The shell thickness of the hollow carbon spheres is observed to be 10 nm, which provide high surface area in the catalysts. The elemental mappings in Fig. 3(a) confirm the existence of FeNi nanoparticles and uniform distribution of N and C elements in the FeNi@NHCS structure. The strong N signals in the EELS mapping indicated the high-concentration doping of N in the porous carbon, which will result in abundant nitrogen containing bonds. A magnified STEM survey in Fig. 3(b) given a more detailed structure of the FeNi nanoparticles on the NHCS. It's observed that all of the FeNi nanoparticles are covered by a loose N–C shell. Interestingly, the distribution of the Fe, Ni atoms in the alloyed particles are not homogenous, but consist of Ni-rich core with Fe-rich shell. The unique structure of the FeNi NPs could be contributed to the different decomposition temperatures of iron chloride and nickel chloride. During the pyrolysis process, nickel chloride decomposed first and then the reduced nickel atoms preferentially nucleate and crystallize. Subsequently, the reduced Fe grown on the Ni particles to form a core/shell structure. Fig. 3(c) shows one typical FeNi nanoparticle on NHCS, it reveals a thin N–C shell with a thickness of ∼3 nm wrapped on the surface of particles. The clear interface between the FeNi nanoparticles and N–C support suggests that synergistic effect will effectively enhance the electrical conductivity.^12,38^ In Fig. 3(d), the high-resolution TEM image indicates that cubic FeNi phase, respectively. The composition of the FeNi alloy on the NHCS was measured by inductively coupled plasma optical emission spectrometer (ICP-AES). As revealed in Table 1, the atomic ratio of Fe : Ni in the alloy is systematically lower than their stoichiometry ratio, suggesting that the Fe precursors are not fully pyrolyzed at 400 °C in highly separated systems. Such inference was validated by measuring the composition of the sample T1, an atomic ratio of Fe : Ni = 1 : 1.08 was determined for FeNi@NHCS sample prepared at 500 °C. The structure of the prepared FeNi@HCMS samples are shown in Fig. S2(a),† a broad diffraction peak near 25° is indexed to the (002) crystal plane of graphitic carbon, revealing the presence of carbon with certain defects and high graphitization. As shown in Fig. 4(a) and (b), the FeNi@NHCS prepared at different annealing temperatures (samples T0–T4) and Fe/Ni ratios (samples C0–C4). Aside from the carbon peak, all of samples displays three distinct characteristic peaks at 44.2°, 51.5°, and 75.8°, which match well with the (111), (200), and (220) crystal planes of the FeNi alloy(PDF# 38-0419), respectively. The results demonstrate the successful deposition of FeNi alloy onto the carbon sphere. Further observation revealed that as the annealing temperature increased, the three diffraction peaks corresponding to the FeNi alloy became sharper. This indicated that higher annealing temperatures led to stronger FeNi alloy crystallization and more evident particle agglomeration. Similarly, increasing the proportion of Ni in the initial metal source resulted in better crystallinity of the prepared FeNi alloy. The pore structure and specific surface area of the HCMS and FeNi@HCMS samples were further analysed by N_2_ sorption measurements. As shown in Fig. 4(c), the adsorption and isotherms of NHCS and a typical FeNi@NHCS sample (R2) exhibited noticeable hysteresis when *P*/*P*_0_ is greater than 0.45. Both sample exhibit type-IV curves with distinct hysteresis loops, disclosing the presence of abundant mesoporous pores. Further, both hysteresis loops belong to type-H2, suggesting that the pore size distribution in the mesoporous carbon is quite uniform. The pore size distribution diagram (Fig. 4(d)) reveals that the average pore sizes for sample R2 and NHCS are 4.03 nm and 4.70 nm, respectively, with most pore sizes concentrating at 4 nm. The specific surface areas of NHCS and sample R2 are 1163.961 m^2^ g^−1^ and 546.873 m^2^ g^−1^, respectively. An obvious decrease was observed for the specific surface area, which is attributed to the deposition of FeNi alloy particles occupying some pores on the carbon sphere surface. These results show that the prepared FeNi@NHCS samples still possess considerable surface area and abundant mesoporous pores, which are favourable for mass transfer and will provide more contact interface and active sites to promote the ORR/OER process.

**Table 1:** Details of metal precursors used in FeNi@HCMS, with their compositions determined by ICP-AES; the characteristic electrochemical parameters of FeNi@HCMS samples in KOH solutions

Labels (annealing temperature)	Molar ratio of Fe : Ni	Composition measured by ICP-AES	ORR *E*_1/2_ (V *vs.* RHE)	The initial potential (V *vs.* RHE)	Limiting current density (mA cm^−2^)	OER overpotential (mV)
T0 (400 °C)	1 : 1	1 : 1.41	0.793	0.945	6.12	275
T1 (500 °C)	1 : 1	1 : 1.08	0.735	0.848	5.68	278
T2 (600 °C)	1 : 1	—	0.726	0.845	4.64	307
T3 (700 °C)	1 : 1	—	0.732	0.858	4.22	331
T4 (800 °C)	1 : 1	—	0.741	0.849	4.69	334
R0 (T0)	1 : 1	1 : 1.41	0.793	0.945	6.12	275
R1 (400 °C)	1 : 2	1 : 2.71	0.807	0.983	4.61	294
R2 (400 °C)	1 : 3	1 : 4.00	0.828	0.99	5.47	281
R3 (400 °C)	1 : 4	1 : 6.37	0.811	0.976	4.83	283
R4 (400 °C)	1 : 5	1 : 7.39	0.810	0.978	5.21	304
Pt/C	—	—	0.90	1.03	5.67	—
RuO_2_	—	—	—	—	—	300

**Fig. 5:**
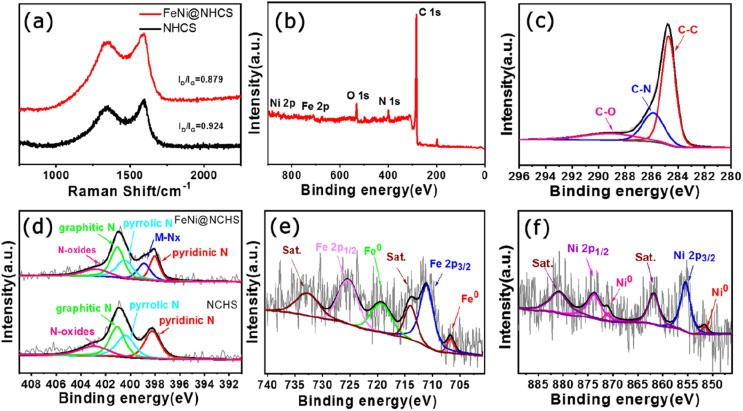
(a) Raman spectra of NHCS and the sample R2; (b) XPS spectra of the sample R2; high resolution XPS spectra of (c) C 1s, (d) N 1s, (e) Fe 2p, (f) Ni 2p.

Raman spectra of NHCS and sample R2 (Fig. 5(a)) showed that both have two prominent peaks located at 1350 cm^−1^ and 1590 cm^−1^, representing the D band and G band of carbon, respectively. The D band corresponds to disordered sp^2^ hybrid carbon, reflecting the degree of defects in the lattice of carbon atoms. The G band represents the expansion peak of sp^2^ carbon, indicating the presence of crystalline graphitic carbon.^40^ Comparing the peak intensities of the D bands and G bands of NHCS and sample R2, the value of *I*_D_/*I*_G_ decreased from 0.924 to 0.879, demonstrating that the growth of FeNi alloy on NHCS surface further promoted the graphitization of carbon, which help to improve the electrical conductivity and accelerate electron transfer in the catalytic reaction process.

To investigate the valence states and surface chemical state of the samples, X-ray photoelectron spectroscopy (XPS) was employed. The full spectrum of XPS showed the presence of Fe, Ni C, N, and O elements in sample R2 (Fig. 5(b)). The XPS high-resolution spectrogram of C 1s (Fig. 5(c)) can be decoupled to three main peaks located at 284.7 eV, 285.8 eV and 289.1 eV, which are attributed to the referenced C–C, C–N, and C–O species. Five peaks of 398.1 eV, 389.9 eV, 400.4 eV, 401.0 eV, and 402.8 eV were separated by fitting the N 1s spectrogram of sample R2, which correspond to pyridinic N, elemental N, pyrrolic N, graphitic N, and oxidized N, respectively.^40^ It's observed that the as-prepared N-doped carbon support contains abundant pyridine nitrogen and graphitic nitrogen, among which pyridine nitrogen is help to promote the catalyst's conductivity, improve surface wettability, and effectively enhance the electrocatalytic activity of ORR.^42^ On the other hand, graphitic nitrogen has been proven to increase the limiting current density and promote the occurrence of a 4-electron transfer process.^41,43^ Hence, the abundant pyridine nitrogen, graphitic nitrogen, and metal nitrogen in our FeNi@NHCS samples can effectively improve the ORR/OER activity. Fig. 5(e) showed the spectrum of Fe 2p, the existence of Fe^0^ and Fe^3+^ was confirmed according to the appearance of Fe^0^ 2p_3/2_ (706.7 eV), Fe^0^ 2p_1/2_ (719.3 eV), Fe^3+^ 2p_3/2_ (711.0 eV), and Fe^3+^ 2p_1/2_ (725.3 eV) with two satellite peaks (714.0 eV and 732.7 eV), respectively. Similarly, the Ni 2p spectrum was fitted to four peaks, corresponding to Ni^0^ 2p_3/2_ (851.8 eV), Ni^0^ 2p_1/2_ (871.0 eV), Ni^2+^ 2p_3/2_ (855.4 eV), Ni^2+^ 2p_1/2_ (873.7 eV), and two satellite peaks (861.8 eV and 880.8 eV), respectively (Fig. 5(f)). Together with the abundant pyridine nitrogen in Fig. 5(d), the XPS spectra provide evidence for the Fe–N_*x*_/Ni–N_*x*_ coordination in the FeNi@NHCS sample, suggesting strong coupling between FeNi alloy nanoparticles and nitrogen-doped carbon carriers.^33^ The results are consistent with recent reports that the pyridine nitrogen form metal–N active sites (Fe–N_*x*_, Ni–N_*x*_) through the coordination of the metal–N–C structure with metal atoms, optimizing the local electronic structure and facilitating the electrochemical reaction.^44,45^

The electrocatalytic activity of FeNi@NHCS samples was investigated as a function of annealing temperature. CV and LSV measurements were performed in a 0.1 M KOH solution saturated with oxygen using a three-electrode system. From the CV curves (Fig. S2(b)†), it is evident that FeNi@NHCS prepared at different annealing temperatures has obvious oxygen reduction peaks, with sample T0 showing the highest reduction potential. This indicates that the annealing temperature of 400 °C is suitable experimental condition to yield the highest electrocatalytic activity in ORR. This result is consistent with the ORR-LSV curves showed in Fig. S2(c).† The initial potential (*E*_onset_), half-wave potential (*E*_1/2_), and limiting current density (*J*_k_) of sample T0 are 0.945 V, 0.793 V, and 6.12 mA cm^−2^, respectively. These values are significantly better than those of FeNi@NHCS samples prepared at other temperatures. The OER-LSV diagram (Fig. S2†(d)) also shows that sample T0 exhibited the lowest initial potential (*E*_*j*=10mA cm^−2^_), confirming the best OER activity at annealing temperature of 400 °C. This is probably due to the agglomeration of the grown FeNi alloy particles is more likely to occur with the increase of annealing temperature, resulting in an uneven distribution of FeNi alloy on the surface of NHCS, thus affecting the electrocatalytic properties of the samples. Additionally, higher annealing temperatures damage the spherical structure of the carbon support (NHCS), also affecting the electrocatalytic activity.

**Fig. 6:**
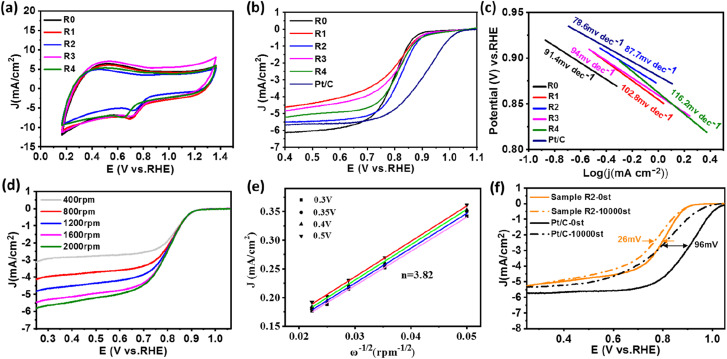
(a) CV curves in O_2_-saturated 0.1 M KOH electrolyte at scan rate of 50 mV s^−1^ of sample (R0–R4); (b) LSV curves in O_2_-saturated 0.1 M KOH electrolyte at scan rate of 5 mV s^−1^ at 1600 rpm, (c) Tafel plots obtained from the corresponding ORR-LSV curves of Pt/C and sample (R0–R4); (d and e) LSV curves of sample R2 at various rotation speeds; the corresponding K–L plots at different potentials. (f) LSV curves of Pt/C and sample R2 before and after stability test.

Under the condition of a constant total molar mass of metal and an annealing temperature of 400 °C, samples with different FeNi components were prepared by changing the atomic ratio of Fe and Ni sources. CV and LSV measurement were also carried out to study the dependence of electrochemical performance on the alloy composition. Fig. 6(a) shows the CV curves of samples R0–R4. Among all LSV curves shown in Fig. 6(b), sample R2 exhibits superior ORR initial potential (*E*_onset_ = 0.99 V) as well as half-wave potential (*E*_1/2_ = 0.828 V) compared with other samples, which is close to those of commercial Pt/C (1.03 V, 0.9 V). Shown in Fig. 6(c) are the Tafel slopes of sample R0–R4 and Pt/C according to the ORR-LSV curve. The Tafel slope of R2 is 87.7 mV dec^−1^, which is the lowest value among the FeNi@NHCS samples and close to that of 20% Pt/C (78.6 mV dec^−1^), indicating better reaction kinetics. It was also attempted to increase the composition of Fe in FeNi alloy in the synthesis (*e.g.* Fe_2_Ni, Fe_3_Ni). However, the pyrolytic reduction yielded mixture of elemental Fe and FeNi alloy, and electrochemical measurements indicated that the mixture exhibited inferior ORR and OER properties compared with FeNi_*x*_ samples. To further understand the ORR catalytic mechanism of sample R2, LSV curves were measured at different rotational rates ranging from 400 to 2000 rpm, which is displayed in Fig. 6(d). Due to the decreased diffusion distance of O_2_ in KOH solution at higher rotational speeds, the current density of sample R2 increases with the increase of rotational speed, indicating a first-order reaction toward oxygen reduction.^46^ The fitted Koutecky–Levich (K–L) curves given in Fig. 6(e) reveals that the number of electron transfers is 3.82. The slope at different potentials shows a good linear relationship, proving that the reduction of oxygen is a first-order reaction kinetic process dominated by a four-electron ORR pathway.^47^ This observation also provides evidence for the excellent reaction kinetics of the FeNi@NHCS sample. The superior performance of the FeNi@NHCS can be attributed to the abundant active sites formed by highly dispersed FeNi alloy on the surface of hollow carbon spheres. Further, the strong coupling of the encapsulated FeNi alloy nanoparticles and the N-doped carbon shell is another contributing factor. This combination of features results in enhanced electrocatalytic activity for ORR, enabling it a promising catalyst for different applications, including fuel cells and metal–air batteries.

To evaluate the stability of the FeNi@NHCS in ORR, continuous cyclic voltammetry testing was conducted on sample R2 in 0.1 M KOH solution between 0.7 V to 1.0 V (*vs.* RHE). As shown in Fig. 6(f), after 10 000 cycles of testing, the limiting current density of sample R2 remains almost unchanged, and the half-wave potential only experiences a minor attenuation of 28 mV. This performance is significantly better than that of the commercial Pt/C catalyst, which exhibits considerable attenuation of 96 mV in the half-wave potential. The results demonstrate the superior stability of FeNi@NHCS for ORR.

**Fig. 7:**
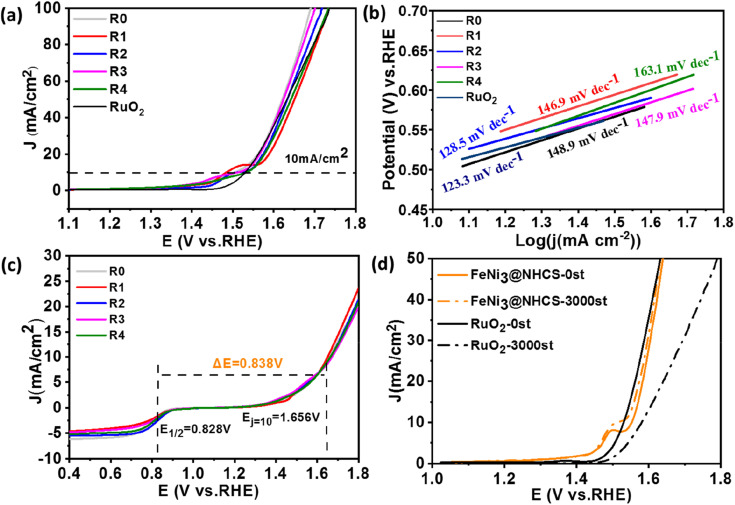
(a) LSV curves of RuO_2_ and samples (R0–R4) in O_2_-saturated 1 M KOH at scan rate of 5 mV s^−1^ at 1600 rpm and (b) Tafel plots; (c) over all LSV curves in O_2_-saturated 0.1 M KOH of sample (R0–R4); (d) LSV curves of RuO_2_ and sample R2 before and after stability test.

The OER behavior of FeNi@NHCS samples was tested in a 1 M KOH solution. The Tafel slope and overpotential (*E*_*j*=10mA_) are effective measurements of the OER performance of the catalysts. From the OER-LSV curve (Fig. 7(a)), it's observed that there is no significant deviation in the OER performance of FeNi@NHCS catalysts with different compositions. Interestingly, samples R0 (275 mV), R1 (294 mV), R2 (281 mV), and R3 (283 mV) exhibit lower overpotentials at 10 mA cm^−2^ than that of commercial RuO_2_ (301 mV), indicating the excellent OER activity of the FeNi@NHCS catalysts. Based on the Tafel slopes derived from the LSV curve (Fig. 7(b)), sample R2 exhibits the lowest Tafel slope (128.5 mV) and is close to commercial RuO_2_ (123.3 mV). This further confirms the superior OER reaction kinetics of the as-prepared FeNi@NHCS samples. Similarly, the remarkable OER properties of FeNi@NHCS materials are also attributed to the large specific surface area of the catalyst, facilitating mass transfer and exposure of active sites. Simultaneously, the abundant active sites and the synergistic interaction of FeNi alloy with the nitrogen-doped carbon support (M–N–C structure) further enhances its electrocatalytic activity. Moreover, the potential difference between ORR and OER (Δ*E*) in KOH (0.1 M) is commonly used to evaluate the overall bifunctional activity of the catalysts, where Δ*E* = *E*_*j*=10 mA_ − *E*_1/2_.^48,49^ A lower Δ*E* value represent more ideal bifunctional catalytic activity. The results in Fig. 7(c) reveal that sample R2 has the lowest Δ*E* (0.838 V) compared to the other samples. Additionally, Fig. 7(d) demonstrates the high OER stability of sample R2, with a 24 mV voltage decay after 3000 cycles, which is significantly lower than that of RuO_2_ (50 mV). The electrochemical properties of the optimized FeNi_*x*_@NHCS sample were compared with recently reported FeNi electrocatalysts and the results are labelled in Table S1.† It reveal that the FeNi_*x*_@NHCS composites in our studies possess some advantages and are potential for the application in rechargeable zinc–air batteries.

**Fig. 8:**
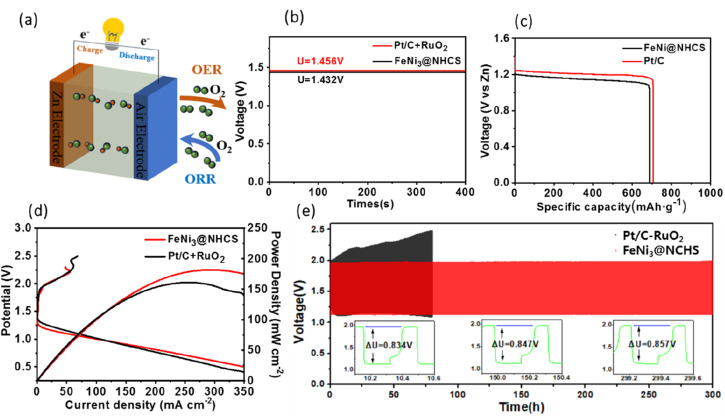
(a) Schematic diagram of the FeNi@NHCS zinc–air; (b) open curve; (c) specific capacity profiles of ZABs at the current density of 10 mA cm^−2^; (d) the discharge polarization curves and power density curves; (e) cycling performance of ZAB based on the sample R2 and the commercial Pt/C–RuO_2_ (10 mA cm^−2^).

The superior bifunctional ORR–OER electrocatalytic activity and electrochemical stability of FeNi@NHCS (R2) prompted us to assemble a ZAB using an electrode composite matrix, as depicted by the schematic diagram shown in Fig. 8(a). The assembled ZAB used FeNi@NHCS electrocatalyst as the air cathode, a zinc sheet as the anode, and solution of 6 M KOH with 0.2 M Zn(Ac)_2_ as the electrolyte. For comparison, ZABs were also assembled with commercial Pt/C–RuO_2_ as the air electrode. Our results demonstrate that sample R2 generates an open-circuit voltage of 1.432 V, being close to that of commercial Pt/C–RuO_2_ (1.456 V) (Fig. 8(b)). Additionally, the specific capacity of ZAB with sample R2 was calculated to be 714 mA h g^−1^, according to the consumed Zn anode. This value is higher than that of Pt/C–RuO_2_ (695 mA h g^−1^), indicating a high utilization of the Zn anode (Fig. 8(c)). Moreover, the corresponding power density curve, derived from the charge–discharge polarization diagram, shows that sample R2 yields a power density of 181.8 mW cm^−2^, which is higher than that of commercial Pt/C–RuO_2_ (161.1 mW cm^−2^). Additionally, it's charging polarization curve is very close to that of commercial Pt/C–RuO_2_, indicating superior charge and discharge performance of the as-made ZABs (Fig. 8(d)). To further explore the stability of the FeNi@NHCS based ZAB (sample R2), we conducted a long-term galvanostatic charge–discharge cycles test at 10 mA cm^−2^, with each cycle taking 20 minutes. The results are presented in Fig. 8(e). The initial discharge and charging potential of the ZAB assembled with sample R2 were 1.134 V and 1.96 V, respectively, while the charge–discharge voltage gap (Δ*U*) was 0.834 V. After 150 h of charge–discharge cycle testing, the discharge and charging potentials were 1.131 V and 1.977 V, respectively, with a Δ*U* of 0.847 V, which represents only a 1.6% decrease compared to the initial Δ*U*. After 300 h of charge–discharge cycle testing, the discharge and charging potentials were 1.13 V and 1.987 V, respectively, with a Δ*U* of 0.857 V, showing a slight decrease of 2.7% compared to the initial Δ*U*. In contrast, the Δ*U* of commercial Pt/C–RuO_2_ assembled ZAB increased from 0.84 V to 1.35 V (a 60.7% attenuation) after 75 h of charge–discharge cycle testing, demonstrating the excellent cyclic stability and durability of the FeNi@NHCS catalysts. Associate with the unique structure displayed in Fig. 3, the superior stability of the ZAB is highly correlated with the electrocatalytic performance of FeNi@NHCS in alkaline electrolyte, which is eventually attributed to the strong coupling of the encapsulated FeNi alloy nanoparticles and the N-doped carbon shell, preventing the corrosion of the FeNi alloy during the electrocatalytic reaction. These results suggest that, as a non-precious metal bifunctional catalyst, the FeNi@NHCS structure has great potential for development and application in the field of zinc–air batteries.

## Conclusion

In conclusion, this study presents an effective pyrolysis approach to deposit FeNi nanoalloy on nitrogen-doped porous hollow carbon spheres, which displays excellent bifunctional electrocatalytic activities and outstanding stability under alkaline environment. The best electrocatalytic performance was achieved in FeNi@NHCS sample prepared at 400 °C with alloy composition of Fe_20_Ni_80_, showing a *E*_1/2_ of 0.828 V in ORR and an overpotential of 281 mV (*vs.* RHE) at 10 mA cm^−2^ in OER. The assembled FeNi@NHCS ZABs has a maximum power density of 181.8 mW cm^−2^ and show only 2.7% decrease in Δ*U* after 300 h charge–discharge cycle test. The excellent electrocatalytic properties were attributed to the mesoporous carbon matrix with large specific surface area, which facilitate mass transfer and exposure of active sites. This study developed a green, durable, and efficient non-noble metal bifunctional catalyst preparation scheme. Meanwhile, it provides a new insight into rationally designing metal–carbon hybrid nanostructures for efficient rechargeable zinc–air battery.

## Conflicts of interest

There are no conflicts to declare.

## Supplementary Material

RA-014-D3RA08572D-s001
