# Supplementary material for: FeNi decorated nitrogen-doped hollow carbon spheres as ultra-stable bifunctional oxygen electrocatalyst for rechargeable zinc–air battery with 2.7% decay after 300 hours cycling
Source: RSC Adv. 2024 Jan 25;14(6):3857–66. doi: 10.1039/d3ra08572d (PMC10810229; doi:10.1039/d3ra08572d)
Supplement: RA-014-D3RA08572D-s001 [file RA-014-D3RA08572D-s001.pdf]

Electronic Supplementary Material (ESI) for RSC Advances.
This journal is © The Royal Society of Chemistry 2024

Detail of Structural Characterization

Raman spectroscopy was performed on the pure NHCS and FeNi@NHCS samples. The morphology of the sample was observed by Field emission scanning electron microscopy (SEM, JSM-7100F) and the structure was analyzed by X-ray diffraction (XRD, Bruker D8) The fine structure and elemental distribution of the FeNi@NHCS were characterized by scanning transmission electron microscopy (STEM, JEOL ARM 200F). The composition of the FeNi alloy on HMCS was tested by inductively coupled plasma optical emission spectrometer (ICP-AES, optimal8000). Nitrogen adsorption/desorption tests were conducted on the pure NHCS and FeNi@NHCS samples. The specific surface area and pore size distribution of the samples were calculate by using Brunauer-Emmett-Teller (BET) method and Barrett-Joyner-Halenda (BJH) model. X-ray photoelectron spectroscopy (XPS) measurement was performed to analyze the valence state and electron structure of the FeNi@NHCS sample.

Calculation of electron transfer number (n)

The Koutecky-Levich equation was used to calculate the electron transfer number (n) per oxygen molecule during ORR process:

$$\frac{1}{j} = \frac{1}{j_L} + \frac{1}{j_K} = \frac{1}{B\omega^{1/2}} + \frac{1}{j_k}$$

$$B = 0.62nFC_{O_2}D_{O_2}^{2/3}v^{-\frac{1}{6}}$$

In the Koutecky-Levich equation, j is the measured current density, j_K and j_L are the kinetic diffusion current density and the kinetic limiting current density, respectively. ω is the angular velocity, n is the transferred electron number, F is the Faraday constant (96,485 C mol^{-1}), C_{O_2} is the saturated concentration of O_2 in 0.1 M KOH (1.2 × 10^{-6} mol cm^{-3}), $D_{O_2}$is the diffusion coefficient of O_2 in 0.1 M KOH (1.9 × 10^{-5} cm^2 s^{-1}), and ν is the kinetic viscosity of 0.1 M KOH (0.01 cm^2 s^{-1}).

Table S1. The electrochemical properties of the optimized $FeNi_x$@NHCS sample compared with recently reported FeNi electrocatalysts.

**Catalysts**	**$E_{1/2}$ (V vs. RHE)**	**$E_{10\ mA\ cm^{-2}}$-1.23V (mV)**	**Reference**
FeNi3@NHCS	0.828	281	This work
Fe/Ni-NC‖FeNi@G	0.885	273	Electrochimica Acta **2023**, 458, 142594
FeNi/N-GPCM	0.883	310	*Separation and Purification Technology* **2022**,308, 122974
FeNi@NC	0.878	360	*International Journal of Hydrogen Energy* **2022**,47, 16025-16035
FeNi@NCSs	0.84	318	*Journal of Colloid and Interface Science* **2022**, 628, 499-507
FeNi-NPC HT	0.859	321	*Journal of Energy Chemistry* 2023,83, 264-274
B-FeNi-NC-1000	0.9	283	*Chemical Engineering Science* **2022**,247, 117038
FeNi Sas/NC	0.84	270	*Advanced Energy Materials* **2021**,11, 2101242
FeNi-NCS-2	0.867	395	*International Journal of Hydrogen Energy* **2021**,47, 984-992
Glu-NiFe	0.85	440	*Electrochimica Acta* **2022**,428, 140938
FeNi/N-CNT	0.82	357	*Journal of Materials Chemistry A* **2022**,10, 9911-9921

Figure S1

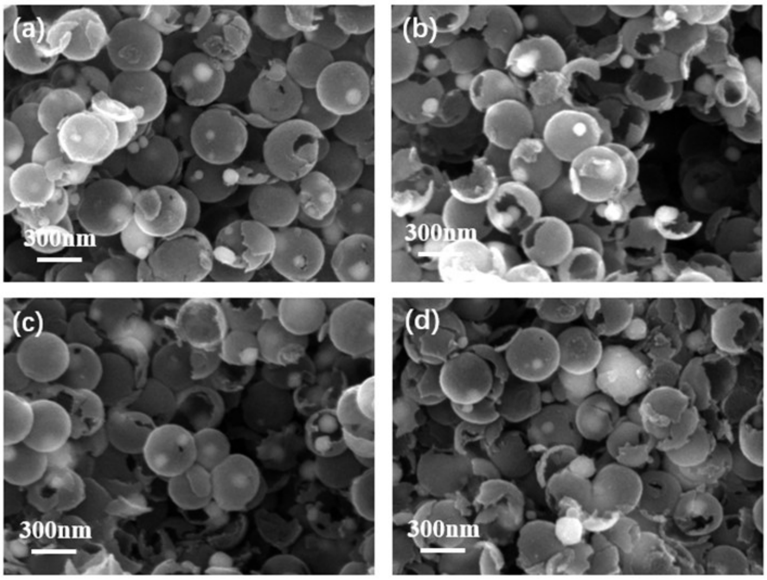

Figure S1. SEM images of FeNi@NHCS samples prepared at (a) 500 ℃, (b) 600 ℃, (c) 700 ℃ and (d) 800 ℃.

Figure S2

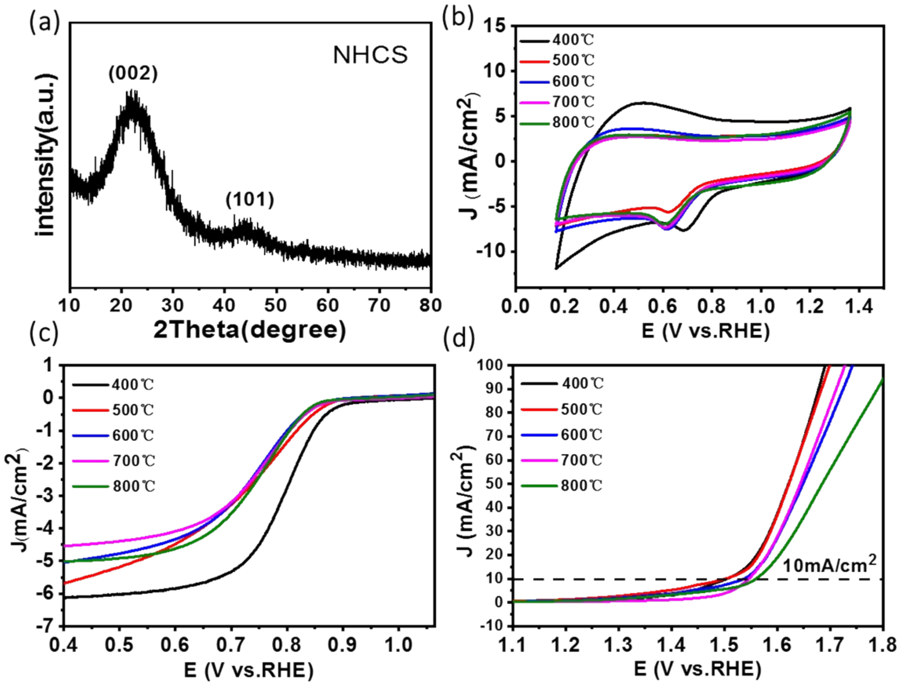

Figure S2. (a) XRD patterns of Pure NHCS sample, (b) C-V curves and (c) LSV curves of sample (T0~T4) in O_2-saturated 0.1 M KOH electrolyte at scan rate of 5 mV s^{-1} at 1600 rpm, (d) LSV curves in O_2-saturated 1 M KOH at scan rate of 5 mV s^{-1} at 1600 rpm of sample (T0~T4).
